# Daily Stress Processes as Potential Intervention Targets to Reduce Gender Differences and Improve Mental Health Outcomes in Mid- and Later Life

**DOI:** 10.1007/s11121-022-01444-7

**Published:** 2022-10-13

**Authors:** Robert S. Stawski, Kelly E. Cichy, Dakota D. Witzel, Ashley C. Schuyler, Madeline J. Nichols

**Affiliations:** 1 Institute of Public Health and Wellbeing, and School of Health and Social Care, University of Essex, Colchester, England UK; 2 Human Development and Family Studies, School of Lifespan Development and Educational Sciences, Kent State University, Kent, OH USA; 3 Center for Healthy Aging, The Pennsylvania State University, State College, PA USA; 4 School of Social and Behavioral Health Sciences, Oregon State University, Corvallis, OR USA

**Keywords:** Depression, Gender, Daily stress, Risk factors, Midlife

## Abstract

The current study examines daily stress processes as risk factors for comprised mental health in midlife and later life, specifically for gender differences in depression risk. Using data from the Midlife in the United States (MIDUS) study and the National Study of Daily Experiences (NSDE), we examine (1) gender differences in depression; (2) the prospective effects of differential exposure and affective responses on 10-year depression status; (3) gender differences in daily stress-depression links. Furthermore, we explore whether the protective factor of help-seeking behavior moderates the effects of daily stress on depression. Participants included 1289 (mage = 55; SD = 12; range = 34–83; 56% female) individuals who completed the second waves of MIDUS and the 8-day NSDE daily diary protocol and participated in the third wave of MIDUS approximately 10 years later. Respondents completed assessments of depression and their seeking assistance from a psychiatrist, mental health professional, counselor, or religious leader. Covariate-adjusted logistic regression analyses revealed increased odds of depression among women compared to men, but no significant gender difference after taking daily stress into account. Higher levels of stressor exposure, negative affect, and affective reactivity were associated with increased odds of depression for both men and women. Compared to those who did not engage in help-seeking behavior, those who did had significantly greater odds of depression, and there were asymmetric patterns of daily stress effects across groups. These findings highlight differential exposure, negative affect, and affective responses to daily stress as potentially accessible intervention targets for reducing stress in daily life and mitigating longer-term depression risk during mid- and later life.

**Supplementary Information:**

The online version contains supplementary material available at 10.1007/s11121-022-01444-7.

Gender differences in mental health, including major depressive disorder and symptomatology in midlife and later life, are well documented, with women twice as likely as men to experience depression (Acciai & Hardy, 2017). Furthermore, the prevalence of major depressive disorder for adults over the age of 50 is 5.4%, with a higher prevalence among women (10.5%) compared to men (6.2%; National Institutes of Mental Health, 2020). Despite theoretical explanations citing differential stressor exposure and reactivity as contributors to gender differences in depression risk (Kuehner, 2017), less research has examined daily stress processes contributing to gender differences in mental health across midlife and later life, despite their potential utility as intervention targets (Smyth et al., 2018, 2022).

## Daily Stress and Its Import for Health

Stressor exposure refers to the frequency of stressors experienced over some interval (e.g., proportion of days; Almeida, 2005). Affective responses, including reactivity and residue, refer to within-person stressor-related changes in affect, albeit over different time scales. Affective reactivity reflects changes in affect associated with stressors reported on the same day, whereas affective residue reflects changes in affect associated with stressors reported on the previous day (Witzel & Stawski, 2021). Thus, reactivity reflects a more proximal effect of stressors on affect, whereas residue reflects a comparatively prolonged effect. While affective reactivity is prospectively associated with worse mental health (Zhaoyang et al., 2020), theoretical models of stress and long-term health posit both more immediate and prolonged stress responses as putative mechanisms driving ill-health (Smyth et al., 2013). No research, however, has considered exposure, reactivity, and residue simultaneously, leaving key insights about the unique daily stress process dimensions contributing to mental health outcomes largely unknown.

### The Relevance of Daily Stress for Health and Prevention During Midlife and Later Life

Reviewing risk and protective factors is critical to the prevention science research cycle (Lich et al., 2013), with recent research highlighting within-person approaches (e.g., daily diary studies) and fluctuations in daily wellbeing (e.g., stress and affect) as providing novel and important insights for prevention research (Linden-Carmichael et al., 2022). This investigation seeks to move beyond more descriptive approaches detailing stress as a risk factor for compromised mental health to identify specific daily stress process components conferring risk. While chronic stress and life events are known contributors to mental health and disparities therein (Kessler, 1997), such sources of stress present conceptual and logistical challenges for intervention and prevention (e.g., ill-defined onset and response dynamics). In contrast, daily stressors occur with greater frequency and are often measured via intensive assessment of individuals over short periods of time (e.g., hours and days), affording enhanced opportunities for the assessment of dynamic affective response mechanisms (Smyth et al., 2018). Thus, daily stress processes represent accessible and realistic intervention target(s) for reducing risk and improving outcomes.

While stress is a risk factor for health across the lifespan, theoretical and empirical research supports age-related vulnerability to stress (Almeida et al., 2011). For example, midlife is characterized by changing and emerging relationship demands (e.g., extended support to adult children, support to grandchildren; caregiving for aging parents), requiring recalibration of goals and roles, creating new sources of stress while compromising resources for coping when stressors occur (Infurna et al., 2020). Furthermore, physical transitions (e.g., menopausal transition) co-occur with these changes in family and professional roles, increasing depression risk (Judd et al., 2012). Thus, midlife through later life represents a window of potentially enhanced vulnerability to stress and associated health-related compromise.

Consistent with this, Hill et al. (2015) advanced the National Institute on Aging Health Disparities Research Framework (NIA-HDRF) to inform research, intervention, and prevention efforts to promote health and mitigate disparities in later life. Informed by the NIA-HDRF, we contend that a daily stress approach holds promise for articulating novel, specific, and malleable stress process mechanisms conferring risk to long-term health and health disparities. To be certain, we are not forwarding that daily stress is the singular mechanism or causal agent responsible for health disparities and their persistence during midlife and later life. Research shows numerous life-course factors contribute to influencing mental health (Colman et al., 2014), some of which shape daily stress processes (Almeida, 2005), but may not be amenable to (direct) intervention and/or prevention efforts. Rather, we view a daily stress approach as elucidating a complementary pathway, one that offers specific, accessible, and malleable targets for intervention and prevention, contributing to the onset, maintenance, and exacerbation of health-related compromise and social gradients therein. Furthermore, a daily stress approach can support moving beyond maintenance interventions or tertiary prevention to more primary (universal) prevention approaches (Pössel, 2005).

### Gender Differences in Depression Across the Lifespan

Across the lifespan, women are at heightened risk for depression (Acciai & Hardy, 2017). Studies of gender and depression predominantly consider adolescence or later life, leaving gaps in our knowledge of gender and risks for compromised mental health in midlife. Explanations for gender differences in depression include women’s unequal access to income and education, experiences of sexism, gender-related roles and expectations (Carmel, 2019), as well as psychological processes, such as gender differences in exposure and responses to stressors and rumination (Kuehner, 2017). Systemic forces, such as sexism and income inequalities, are deeply entrenched in society and culture beginning at an early age, making them more resistant to change and difficult to directly address. Daily stress processes represent a more proximal, realistic intervention target that could immediately reduce risk and improve outcomes among those at greatest risk for compromised mental health (Smyth et al., 2018, 2022). Thus, considering the diversity of ways stress manifests in daily life presents unique opportunities to inform and structure intervention and prevention efforts.

### Gender and Daily Stress Processes Predicting Depression

Prior research reveals gender differences in the distribution and prevalence of stressors and stress responses (Husky et al., 2009). Research on stress contributing to gender differences in depression risk largely focuses on chronic stressors (e.g., caregiving), life events (e.g., trauma), and gender discrimination (Carmel, 2019; Kuehner, 2017). While these pathways are well-validated risk factors for depressive symptomatology, recent work suggests that chronic sources and manifestations of stress may, in part, be a result of responses to more stressors in daily life (Smyth et al., 2013).

Throughout mid- and later life, both women and men experience significant personal and professional transitions that shape both stressor exposure and affective responses to stressors (Infurna et al., 2020). Thus, identifying daily stress processes conferring long-term risk for depression, including the extent to which there is symmetry in the effects of stressor exposure, affective reactivity, and residue across genders, is critical. Role transitions in midlife and later life and convergence in social roles may contribute to symmetry across genders in the effects of stressor exposure on depression risk. In contrast, affective reactivity and residue may confer differential, asymmetrical effects on depression risk for women and men, given gender differences in physiological and affective stress responses and rumination (Kuehner, 2017).

### Help-Seeking Behavior as a Moderator of Daily Stress Predicting Depression

Formal help-seeking behavior (HSB), the seeking of assistance in support of mental health problems from health care (e.g., psychiatrist) or non-health professionals (e.g., clergy), is a key indicator of depression severity (Bebbington et al., 2003). As help-seeking reflects problem-focused and planned behavior in support of one’s mental health (Cornally & McCarthy, 2011), it may serve to buffer the adverse effects of daily stress on mental health. Specifically, HSB may endow individuals with skills and strategies for identifying and preemptively avoiding potentially stressful situations and adaptively regulating one’s affect when stressors occur. Thus, HSB may contribute to minimizing the frequency of and/or temper-affective responses to daily stressors, reducing depression risk.

Research shows that HSB for psychological problems is lower among men and decreases with age, with some evidence suggesting gender differences are reduced starting at age 65 (Karlin et al., 2008). Such gender and age trends, however, do not mean that midlife and older adults with mental health concerns are receiving adequate levels of care, regardless of gender. Indeed, despite a lower prevalence of depression and mental health problems more generally during midlife and later life, older individuals with mental health problems are less likely than those who are younger to be receiving treatment (Karlin et al., 2008). Thus, examining HSB as a moderator of daily stress effects on depression risk during midlife and later life stands to enhance the identification of additional potential sources of risk and targets for intervention and prevention.

### Current Study

The current study aimed to answer the following research questions regarding associations among gender, daily stress, HSB, and depression over 10 years across midlife and later life. First, are there gender differences in depression in midlife and later life? We expect depression to be higher among women compared to men. Second, do daily stress processes predict 10-year depression status? We predict that greater affective responsivity (i.e., reactivity and residue) will be associated with an increased depression status. Third, do daily stress processes partially account for gender differences in depression during midlife and later life? We predict that the gender difference in depression status will be attenuated after daily stress processes are considered. Fourth, are there gender differences in the effects of daily stress process components on depression status? We predict daily stress effects to be stronger among women and also explore potential asymmetries in daily stress process components associated with depression status among women and men. Finally, does engaging in HSB moderate daily stress-depression associations? We predict that engaging in HSB will reduce daily stress-depression associations.

## Method

### Participants and Procedure

Data come from the Midlife in the United States (MIDUS; Radler & Ryff, 2010) study and the National Study of Daily Experiences (NSDE; Almeida, 2005). We drew on the second wave of the NSDE, as well as the second and third waves of the MIDUS, to leverage the enhanced daily well-being assessments and cohort expansion during the second wave. The NSDE consists of a subsample of 2022 MIDUS respondents (*M*_age_ = 56; range = 33–84; SD = 12.20; 54% women) who participated in eight consecutive days of telephone interviews assessing stress, health, and well-being each day from 2004 to 2006. With detailed rationale below, our analytic sample consisted of 1289 individuals who participated in both the NSDE II and MIDUS III; this sample was slightly younger (*M* = 55.20; range = 33–84; SD = 11.45), and 56% women (see Table 1).

**Table 1:** Demographic information pertaining to the total sample and by gender

	Total (*N* = 1289)	Women (*N* = 725)	Men (*N* = 564)
	Mean (SD) or %	Mean (SD) or %	Mean (SD) or %
Age	55.20 (11.45)	55.17 (11.58)	55.24 (11.27)
Employment Status (Working)	53.84	52.55	55.50
Race (White)	92.40	92.55	92.20
Education*			
High school diploma/GED or less	27.54	30.62	23.58
Bachelor’s degree or some college	20.33	50.48	54.26
More than a bachelor’s degree	52.13	18.90	22.16
Marital status (married)*	74.01	68.97	80.50
Stressor frequency (% of days)*	38 (26)	40 (27)	36 (26)
Negative affect (NSD)*	0.11(0.22)	0.12(0.24)	0.10(0.20)
Affective reactivity	0.12(0.22)	0.12(0.22)	0.11(0.21)
Affective residue	0.01(0.16)	0.01(0.17)	0.01(0.14)
Depression @ M2*	11.71	14.90	7.62
Depression @ M3*	11.40	13.38	8.87
Help-seeking behavior @ M2	28.88	30.06	27.34
Help-seeking behavior @ M3	19.90	21.53	17.81

*SD* represents the standard deviation, *NSD* non-stressor day, *M* MIDUS

*represent significant gender differences (*p* < 0.05)

### Measures: MIDUS Data Collection

#### Depression

At both the second and third waves of MIDUS, participants reported on seven items based on DSM-III criteria for depressed affect (Wang et al., 2000). Depression was classified if: (1) participants had endorsed that they had felt sad, blue, or depressed for two weeks or more in a row, that the feelings lasted all or most of the day, and that they felt this way every day or almost every day during the 2 weeks in question; (2) participants had to endorse experiencing at least four additional symptoms during those two weeks (e.g., trouble concentrating, trouble falling asleep). A dichotomous variable was created to indicate depression.

#### Help Seeking Behavior (HSB)

As part of the second wave of the MIDUS survey, participants were asked the number of times they reported seeing a mental health professional in the past 12 months. Specific categories of professionals included: (a) psychiatrist, (b) general practitioner or other medical doctors, (c) psychologist, professional counselor, marriage therapist, or social worker, and (d) minister, priest, rabbi, or other spiritual advisors. A dichotomous HSB variable was created to reflect whether respondents indicated any engagement across the four categories.

#### Gender

Participants were asked to indicate whether they identified as female or male while completing the survey during the second wave of MIDUS.

## Daily Measures: NSDE Data Collection

### Daily Negative Affect (NA)

Negative affect was assessed using a scale developed for the NSDE (see Scott et al., 2020 for items and psychometric overview), consisting of 14 items asking, “How much of the time today did you feel …” using a 5-point Likert scale (0 = none of the time; 4 = all of the time). Responses were averaged with higher scores representing higher negative affect (*α*_within-person_ = 0.77, *α*_between-person_ = 0.97).

### Daily Stress

The Daily Inventory of Stressful Experiences (DISE) was utilized to measure frequency, exposure, and type of daily stressors experienced (Almeida et al., 2002). Participants are asked if a specific type of negative event occurred within the last 24 h (0 = no, 1 = yes). These specific types of events included arguments, avoided arguments, work overloads, home overload, or network stressors (i.e., stressors that occur to a close friend or relative that are stressful for the respondent). Days were coded dichotomously to reflect whether any of the types of events were reported.

### Covariates

Based on previous research regarding daily stress and affective reactivity associations (Stawski et al., 2019a, b), we additionally covaried for age (continuous) at NSDE II, marital status (married; not married), employment status (working; other), race (White; other), and level of education (less than or equivalent to high school diploma; bachelor’s degree or some college; more than a bachelor’s degree). All covariates were obtained from the second wave of MIDUS.

### Analytic Strategy

#### Daily Stress Process Indicators and Data Reduction

Stressor exposure represented the proportion of study days participants reported any stressors. Following previous research (Stawski et al., 2019a, b; Witzel & Stawski, 2021), affective reactivity and residue slopes were derived from individual-specific multiple regressions with same- and previous-day stressor occurrence predicting negative affect. Thus, affective reactivity and residue are operationally defined as time-varying stressor-affect associations, quantifying changes in affect associated with a stressor occurring on the same and previous day, respectively. This model parameterization also yields an intercept reflecting a negative effect on non-stressor days, which was retained for analyses. The estimates of stressor exposure, non-stressor day negative affect, affective reactivity, and affective residue were used as the operant daily stress process indicators for predicting depression.

Individuals reporting stressors on either 0 (*n* = 252) or 100% (*n* = 70) of days were excluded from analyses as reactivity and residue slopes could not be computed for these individuals. Additionally, 411 participants who participated in the NSDE but did not have follow-up data from MIDUS 3 were excluded, resulting in a final analytic sample of 1289 respondents. Compared to our analytic sample, those who attrited were significantly older, non-white, less likely to be married and working, had lower levels of education, less frequent stressor exposure, and higher daily negative affect (all *p*s < 0.05). The samples did not differ in gender, depression, HSB, affective reactivity, or affective residue.

#### Gender, Daily Stress, and Help-Seeking Behavior Predicting Depression

Depression status at MIDUS 3 was modeled using logistic regression in SAS v9.4. For primary analyses of gender differences, our first model considered gender differences in depression (model 1). Next, model 1 was expanded to include the daily stress process variables–stressor exposure, negative affect on non-stressor days, reactivity, and residue (model 2). Finally, model 2 was expanded to add four interaction terms, allowing for gender differences in the daily stress effects (model 3). For integrating HSB, our initial model considered the main effects of HSB and the four daily stress process indicators (model 1) and was expanded to add HSB by daily stress interactions (model 2). Analysis of simple slopes by gender and HSB were conducted using ESTIMATE commands in PROC LOGISTIC and analyses were stratified by group. Covariates were included in all models. Daily stress process variables were standardized (*z*-scores), so slope estimates were centered at the sample mean and reflect the change in odds per standard deviation. Statistical significance was evaluated using *α* = 0.05, evaluating the odds ratio and associated 95% confidence interval.

## Results

### Descriptive Statistics

As shown in Table 1, 11.7 and 11.4% of the sample met the criteria for depressive disorder when assessed at MIDUS 2 and 3, respectively. HSB was reported by 28.9% of the sample at the MIDUS 2 assessment. For daily stress process components, participants reported experiencing stressors on 38% of days (SD = 26%), and low levels of negative affect (NA) on non-stressor days (*M* = 0.11, SD = 0.22). Affective reactivity was 0.12 (SD = 0.22) on average, indicating that NA doubled on stressor days compared to non-stressor days, while affective residue was 0.01 (SD = 0.16), suggesting small increases in NA sustaining to the day after a stressor was reported. Importantly, each daily stress process indicator exhibited considerable variation. Gender differences were selected, with a greater percentage of women meeting criteria for depressive disorder (*p* = 0.01), having more frequent stressor exposure (*p* = 0.004), and higher levels of NA on non-stressor days (*p* = 0.04).

### Gender and Daily Stress Processes Predicting Depression

Women had significantly greater odds of depression (OR = 1.46, *p* = 0.04; Table 2–model 1). In model 2 (Table 2), the gender difference was reduced and no longer significant (OR = 1.34, *p* = 0.13) when daily stress variables were added to the model. Furthermore, more frequent stressor exposure (OR = 1.36, *p* < 0.05), higher NA on non-stressor days (OR = 1.34, *p* < 0.001), and greater affective reactivity (OR = 1.33, *p* < 0.001) were each associated with increased odds of depression, while affective residue was marginally so (OR = 1.16, *p* = 0.08).

**Table 2:** Logistic regression results of gender differences and daily stress processes predicting MIDUS Wave 3 depression

	Model 1	Model 2	Model 3a–men	Model 3b–women	
	OR [%95 CI]	OR [%95 CI]	OR [%95 CI]	OR [%95 CI]	*P* gender difference
Gender (female = 1)	1.46** [1.01, 2.11]	1.34 [0.92, 1.96]	-	-	
Stressor exposure		1.36*** [1.11, 1.66]	1.35 [0.96, 1.89]	1.38*** [1.08, 1.78]	0.91
Negative affect (NSD)		1.34*** [1.14,1.57]	1.26 [0.96, 1.66]	1.39*** [1.12, 1.71]	0.60
Affective reactivity		1.33*** [1.14,1.54]	1.13 [0.84, 1.52]	1.46*** [1.21, 1.77]	0.15
Affective residue		1.16* [0.99,1.36]	1.09 [0.78, 1.51]	1.18* [0.97, 1.43]	0.69

All models covary for age, education, marital status, employment status, and race

*p < 0.10, **p < 0.05, ***p < 0.01

### Gender Differences in Daily Stress Processes Predicting Depression

Next, we included interactions to examine gender differences in daily stress process components predicting depression. While the main effects of stressor exposure, NA on non-stressor days, and affective reactivity were significant (all *p*s < 0.01), none of the interactions were statistically significant (all *p*s ≥ 0.15). Analysis of simple slopes and stratified analyses revealed positive associations among each of the daily stress variables and depression for both men (Table 2–model 3a) and women (Table 2–model 3b). The effects of stressor exposure, NA on non-stressor days, and affective reactivity were only significant among women (all *p*s ≤ 0.01).

### Exploratory Analyses of Daily Stress Processes Effects by Stressor Type

As all analyses heretofore collapsed across different types of stressors to reflect exposure and affective response associated with any stressors, we conducted exploratory analyses examining exposure, affective reactivity, and residue associated with each of the five types of stressors represented in the DISE (Supplemental Table 1). As shown in Supplemental Table 2, more frequent avoided arguments (OR = 1.24, *p* = 0.03) and home overloads (OR = 1.20, *p* = 0.05) were associated with increased odds of depression. Increased affective reactivity associated with arguments (OR = 1.35, *p* < 0.001) and home overloads (OR = 1.18, *p* = 0.03), and increased residue associated with avoided arguments (OR = 1.23, *p* = 0.01) were associated with increased odds of depression. There were no significant gender differences when effects were disaggregated by stressor type (all *p*s ≥ 0.23). Interestingly, results from the simple slope and stratified analyses revealed select and asymmetric DSP effects predicting depression for men and women. For men, greater affective reactivity associated with arguments (OR = 1.41, *p* = 0.03) and affective residue associated with avoided arguments (OR = 1.53, *p* = 0.01) were associated with increased odds of depression. For women, higher NA on non-stressor days (OR = 1.33, *p* = 0.02) and greater affective reactivity associated with arguments (OR = 1.36, *p* = 0.002) were associated with increased odds of depression.

### Help-Seeking Behavior as a Moderator of Daily Stress Predicting Depression

As shown in Table 3 (model 1), compared to those who did not engage, individuals who reported engaging in HSB had significantly greater odds of depression (OR = 1.85, *p* = 0.002). Similarly, greater exposure (OR = 1.31, *p* = 0.01), NA on non-stressor days (OR = 1.29, *p* = 0.003), and affective reactivity (OR = 1.36, *p* < 0.001) were each associated with increased odds of depression. Adding interactions between HSB and daily stress process variables did not reveal any reliable evidence of moderation effects (all *p*s ≥ 0.26). Results of simple slope and stratified analyses, however, yielded significant, albeit asymmetric daily stress effects across these groups. For individuals who did not engage in HSB (Table 3–model 2a), greater NA on non-stressor days (OR = 1.37, *p* = 0.02) and affective reactivity (OR = 1.40, *p* < 0.001) were associated with increased odds of depression. Among those who did engage in HSB (Table 3–model 2b), greater exposure (OR = 1.50, *p* = 0.02) was associated with increased odds of depression, with NA on non-stressors days (OR = 1.21, *p* = 0.09) and affective reactivity (OR = 1.30, *p* = 0.07) marginally so.

**Table 3:** Logistic regression results of help seeking behavior and daily stress processes predicting MIDUS Wave 3 depression

	Model 1	Model 2a–no HSB	Model 2b–HSB	
	OR [%95 CI]	OR [%95 CI]	OR [%95 CI]	*P* HSB difference
Help-seeking behavior	1.85*** [1.26, 2.71]			
Stressor exposure	1.31*** [1.07, 1.61]	1.17 [0.89, 1.54]	1.50** [1.08, 2.06]	0.26
Negative affect (NSD)	1.29*** [1.09, 1.51]	1.38** [1.06, 1.78]	1.21* [0.97, 1.52]	0.47
Affective reactivity	1.36*** [1.17, 1.58]	1.40*** [1.17, 1.68]	1.30* [0.98, 1.73]	0.67
Affective residue	1.13 [0.96, 1.33]	1.08 [0.88, 1.33]	1.23 [0.94, 1.62]	0.46

All models covary for age, education, marital status, employment status, and race

*p < 0.10, *p < 0.05, **p < 0.01

### Sensitivity Analysis

We re-estimated all models, including depression status at MIDUS 2 as a covariate. While MIDUS 2 depression status was a significant predictor of MIDUS 3 depression status in all models (all *p*s < 0.001), neither the direction nor statistical significance of estimates changed.

### Supplemental Analysis–Daily Stress Predicting Incident Depression

To examine daily stress and incident, or new depression onset, 10 years later, we repeated analyses restricting the analytic sample to those who did not meet the criteria for depression status at MIDUS 2. Greater exposure (OR = 1.35, *p* = 0.02) and affective reactivity (OR = 1.31, *p* = 0.01) were each associated with increased odds of depression (Supplemental Table 2).

## Discussion

The current study yielded several key takeaways. First, multiple daily stress process indices, including differential negative affect, exposure, and affective reactivity to daily stressors, contribute to gender differences in depression. Second, these same indices contribute to predicting increased depression risk, with exposure and affective reactivity specifically associated with incident depression over 10 years. Additionally, covarying for depression status at MIDUS 2 did not alter these results, suggesting that the daily stress effects are not an artifact of extant depression. Third, while gender differences in daily stress-depression links were not significant, results suggest negative affect, exposure, and affective reactivity are potentially viable targets for intervention and prevention of depression for both women and men. Finally, although HSB did not significantly moderate daily stress effects, the pattern of daily stress-depression associations was asymmetric across groups. Specifically, differential negative affect and affective reactivity were predictive of depression among individuals who did not engage in HSB, whereas differential stressor exposure was associated with depression among those who did. Taken together, daily stress processes represent a complex constellation of unique and potent risk factors that represent potentially modifiable targets for intervention and prevention efforts related to depression in midlife and later life.

### Gender and Daily Stress Processes Predicting Depression

Consistent with prior studies, women were significantly more likely than men to experience depression, suggesting women’s heightened risk for depression persists into midlife and later life (Acciai & Hardy, 2017). Importantly, the gender difference in depression was no longer significant after accounting for daily stress processes. We acknowledge that daily stress is not a singular driver of depression but a complementary pathway contributing to depression risk among women and men. Furthermore, our findings provide insights into exposure and affective reactivity as specific daily stress process components contributing to gender differences in depression.

### Gender Differences in Daily Stress Processes Predicting Depression

Contrary to expectations, we did not observe gender differences in daily stress-depression associations. Furthermore, while daily stress effects were in the same direction for women and men, they were only significant among the former. One reason for such findings could be that men have fewer stressor days than women, compromising reliability to detect affective responses and associated individual/group differences therein. Another explanation is that analyses reducing exposure and affective responses to “any” stressors obscure nuances and gendered patterning of daily stress-depression links. Our exploratory analyses support this, as findings revealed symmetries and asymmetries across gender when we considered the specific stressor type or context. Specifically, affective reactivity to arguments was a significant predictor of depression for men and women, whereas affective residue or lasting reactivity associated with avoided arguments was only associated with increased risk for men. In contrast, women’s higher NA on non-stressor days also predicted increased odds of depression. These differences are in line with gender socialization and gendered social roles, where the link between women’s depression risk and higher NA on non-stressor days may reflect imbalanced power structures at work and within families (Carmel, 2019). For men, gendered expectations regarding masculinity, including perceived pressure to be assertive and interpersonally dominant, may contribute to the significance of reactivity to avoiding arguments and to the lasting effects of these specific stressors because not engaging in the disagreement may violate these gendered norms (Hsu et al., 2021).

While reduction of both exposure and affective responses to daily stressors, broadly, stands to reduce depression risk for men and women, the current results highlight the potential efficacy for tailoring intervention and prevention efforts to specific and different types of daily stressors and stress processes. These findings underscore the import, as well as the complexity and diversity, of ways in which naturally occurring stress in daily life contributes to depression. Furthermore, they extend research describing stress as a risk factor for depression and provide nuanced information about the specific aspects of daily stress conferring risk, enhancing utility for prevention science and practice (Lich et al., 2013), particularly when risk factors may present episodically in daily life (Linden-Carmichael et al., 2022).

### Help-Seeking Behavior and Daily Stress-Depression Links

Contrary to expectations, HSB did not moderate daily stress effects, and this could be due to several reasons. First, given that participants’ HSB (over the past 12 months) was assessed as part of the MIDUS survey, at least 3 months prior to their participating in the NSDE, it is unclear if individuals were still engaging in HSB during their participation in the NSDE, or if individuals who were not engaging in HSB had begun doing so. Second, our outcome reflects participants’ meeting the criteria for major depressive disorder. As HSB is highly predictive of depression severity (Bebbington et al., 2003), our observed positive association between HSB and depression is consistent with this. The moderating effects of HSB may be more nuanced, emerging if symptom severity, either among those sub- or supra-threshold for meeting depression criteria, were considered. Finally, reducing HSB to a dichotomous variable, while convenient and affording larger cell sizes, does compromise the nuance of this construct.

While HSB did not significantly moderate daily stress-depression associations, non-stressor levels of negative affect and affective reactivity were associated with increased risk among those not engaged in HSB, whereas exposure was associated with increased risk among those who did seek help. Such patterning is consistent with HSB buffering the effects of daily stress on depression (Cornally & McCarthy, 2011) but also conveys a complex picture of what about daily stress confers risk and for whom. The effect of affective reactivity was specific to those not engaged in HSB, suggesting that individuals receiving help benefit from supports and strategies for reducing the potency of affective responses contributing to depression in later life, highlighting the benefits of formal help for wellbeing in daily life. In contrast, more frequent stressor exposure, but not affective response, was associated with increased risk among individuals who engage in HSB. While eliminating stressors from people’s lives is unrealistic, efforts to reduce their occurrence may be a particularly salient target for those who have/are engaged in formal HSB.

### Considering Patterns of Associations vs. Statistical Significance

Despite a lack of evidence for gender and HSB moderating daily stress-depression associations, additional points regarding the patterning of associations are worth mentioning. While the pattern of daily stress-depression associations was similar for women and men, select asymmetries in associations were revealed when the type of stressor was considered. Similarly, the pattern of daily stress effects was asymmetric between HSB groups. We have shown that the power to detect sample average affective reactivity is quite strong across designs varying in the number of participants and observations per participant, whereas the power to detect individual/group difference moderators with those same designs is comparatively poor (Stawski et al., 2019a, b). Given that the pattern of daily stress-depression associations was similar between women and men as well as HSB groups, despite the statistical significance (or lack thereof) of all and analogous simple slopes, we believe daily stress process components represent candidate risk factors and intervention targets for both genders and HSB groups. Furthermore, (a)symmetries in the significant daily stress components may provide insight into potential prioritization of what to initially target, and for whom, when considering intervention and prevention efforts. Targeting reduction of stressor exposure is likely beneficial for all, regardless of gender or HSB, as affective reactions to daily stressors cannot occur without a catalyst. Additional and more specific targeting, however, may be beneficial based on circumstances. For example, women and men may both benefit from supports for reducing exposure and tempering affective responses to interpersonal stressors (i.e., arguments), with men further benefiting from supports aimed at reducing protracted responses to interpersonal stressors (i.e., residue associated with avoided arguments). One-size-fits-all intervention and prevention efforts may not be maximally efficacious, particularly when dealing with an individual’s lived experiences. Thus, we argue considering the (a)symmetries in the pattern of associations, as opposed to strict reliance on statistical significance, has pragmatic value when weighing the translational value of empirical findings.

### Practical Implications and Applications for Intervention and Prevention Research

Intervention and prevention efforts for reducing stressor exposure and affective reactivity, scalable for practice and use in real-time in daily life, are critical for mitigating stress and promoting well-being in daily life. Research shows stressor forecasting, anticipatory coping (Neupert & Bellingtier, 2019), and deep breathing techniques (Smyth & Heron, 2016) are all associated with reduced stress and improved daily well-being. Additionally, prosocial activities have been particularly beneficial for promoting daily well-being across the adult lifespan during COVID-19 (Sin et al., 2021). Each of these could be skills mental health professionals could help individuals develop and deploy for practice and use in real time. Such skills and strategies could be prompted remotely using mHealth and ecological momentary intervention approaches (Smyth & Heron, 2016) to reduce daily stress.

### Limitations and Future Directions

Although the present study contributes to the critical phase of reviewing risk and protective factors, it is not without limitations. Affective reactivity and residue in the daily diary context are relatively gross in terms of the time course. Complementary designs, such as ecological momentary assessment (EMA), that include more assessments within and across days would enhance the granularity of exposure–response dynamics, offering additional information for intervention and prevention schemes (Smyth et al., 2018). Furthermore, our investigation of the daily stress processes only considers exposure and negative affective responses to stressors, but does not address subjective stressor appraisal or coping, nor stressor-related changes in positive affect, all implicated in depression risk (Rackoff & Newman, 2020). Similarly, although we explored whether daily stress effects were specific to certain types of stressors, such analyses stretch data, even in larger diary studies such as the NSDE, thin. As stressors are not interchangeable, and stressors of the same nominal type can exert different impacts (Witzel & Stawski, 2021), clarifying how stressors, appraisals, and their interactions confer risk for depression is a critical avenue for future research.

While our assessment of HSB allowed for detailing who engaged, our study cannot speak to whether and how such engagement was shaped by predisposing characteristics, enabling resources, and need (Andersen, 1995). Thus, our study sheds light onto what daily stress is associated with depression in the context of HSB (or lack thereof) but cannot disentangle how key factors associated with HSB may influence daily stress processes or vice versa. Finally, the NSDE and MIDUS cohorts are nonrepresentative. Gender response options in MIDUS were binary, meaning the current results may not apply outside this narrow definition. Additionally, the MIDUS sample is well-educated, predominantly white, and lacking racial and ethnic diversity. Furthermore, all data collection took place before the COVID-19 pandemic, which highlighted the salience of mental health and daily well-being. As such, these results may underestimate the impact of daily stress on mental health. To this end, future research exploring daily stress-mental health associations among more diverse populations and in current times stands to make important contributions to promoting mental health, particularly among underrepresented, marginalized, and vulnerable populations.

### Summary and Conclusions

The current study reveals daily stress processes as proximal, accessible, and realistic intervention targets for reducing depression risk and improving mental health outcomes. Higher levels of negative affect, stressor exposure, and affective reactivity in daily life were each associated with increased depression 10 years later among women and men in midlife and later life and accounted for gender differences in depression, highlighting daily stress as a complementary pathway contributing to gender disparities in mental health. Furthermore, our findings revealed unique insights into different dimensions of daily stress that might be targeted for intervention depending on one’s HSB. In sum, this study adds perspective on daily stress as a modifiable risk factor, as well as potential approaches for the identification and articulation of what and for whom intervention and prevention efforts can be maximized in service of promoting mental health during midlife and later life.

## Supplementary Information

Below is the link to the electronic supplementary material.

Supplementary file1 (DOCX 23 KB)
