# Supplementary material for: Daily Stress Processes as Potential Intervention Targets to Reduce Gender Differences and Improve Mental Health Outcomes in Mid- and Later Life
Source: Prev Sci. 2022 Oct 13;24(5):876–86. doi: 10.1007/s11121-022-01444-7 (PMC9558008; doi:10.1007/s11121-022-01444-7)
Supplement: Supplementary file 1 — Supplementary file1 (DOCX 23 KB) [file 11121_2022_1444_MOESM1_ESM.docx]

Supplemental Tables

**Table 1**

*Gender and Daily Stress Processes Predicting Prevalence of Depression in MIDUS Wave 3 (stratified by stressor type)*

|  | Model 1 |  | Model 2 |  | Model 3a - Men |  | Model 3b - Women |  |
| --- | --- | --- | --- | --- | --- | --- | --- | --- |
|  | OR [%95 CI] |  | OR [%95 CI] |  | OR [%95 CI] |  | OR [%95 CI] | p Gender Difference in Model 3 |
| Gender (Female=1) | 1.46* [1.01, 2.11] |  | 1.30 [0.88, 1.93] |  |  |  |  |  |
| Negative Affect (NSD) |  |  | 1.24* [1.04, 1.48] |  | 1.25 [0.60, 1.76] |  | 1.33* [1.06, 1.68] | 0.76 |
| Stressor Exposure |  |  |  |  |  |  |  |  |
| Arguments |  |  | 0.90 [0.74, 1.09] |  | 0.78 [0.52, 1.18] |  | 0.92 [0.73, 1.16] | 0.49 |
| Avoided Arguments |  |  | 1.24* [1.03, 1.49] |  | 1.21 [0.85, 1.73] |  | 1.26^†^ [0.99, 1.59] | 0.84 |
| Work Overloads |  |  | 1.09 [0.92, 1.31] |  | 1.06 [0.79, 1.43] |  | 1.14 [0.90, 1.45] | 0.70 |
| Home Overloads |  |  | 1.20* [1.00, 1.44] |  | 1.29 [0.91, 1.85] |  | 1.13 [0.91, 1.42] | 0.53 |
| Network Stressors |  |  | 1.06 [0.88, 1.27] |  | 1.10 [0.73, 1.67] |  | 1.01 [0.82, 1.25] | 0.72 |
| Affective Reactivity |  |  |  |  |  |  |  |  |
| Arguments |  |  | 1.35** [1.16, 1.59] |  | 1.41* [1.04, 1.93] |  | 1.36** [1.12, 1.64] | 0.82 |
| Avoided Arguments |  |  | 1.04 [0.88, 1.24] |  | 0.75 [0.48, 1.18] |  | 1.16 [0.82, 1.27] | 0.08 |
| Work Overloads |  |  | 1.02[0.87, 1.21] |  | 1.03 [0.77, 1.39] |  | 1.02 [0.82, 1.27] | 0.94 |
| Home Overloads |  |  | 1.18* [1.02, 1.35] |  | 1.33 [0.89, 1.98] |  | 1.14 [0.97, 1.34] | 0.49 |
| Network Stressors |  |  | 0.99 [0.84, 1.16] |  | 1.03 [0.77, 1.37] |  | 0.96 [0.79, 1.15] | 0.69 |
| Affective Residue |  |  |  |  |  |  |  |  |
| Arguments |  |  | 1.06 [0.91, 1.23] |  | 1.07 [0.77, 1.47] |  | 1.07 [0.89, 1.28] | 0.49 |
| Avoided Arguments |  |  | 1.23** [1.06, 1.43] |  | 1.53** [1.13, 2.07] |  | 1.12 [0.92, 1.35] | 0.69 |
| Work Overloads |  |  | 1.01 [0.86, 1.19] |  | 0.94 [0.73, 1.21] |  | 1.09 [0.87, 1.37] | 0.99 |
| Home Overloads |  |  | 1.02 [0.87, 1.21] |  | 0.84 [0.57, 1.24] |  | 1.09 [0.90, 1.32] | 0.09 |
| Network Stressors |  |  | 1.02 [0.88, 1.19] |  | 0.81 [0.53, 1.24] |  | 1.08 [0.93, 1.27] | 0.38 |

*Note***.** All models covary for age, education, marital status, employment status, and race. ^†^p<.10, *p<.05, **p<.01

**Table 2**

*Gender and Daily Stress Processes Predicting (Incident) MIDUS Wave 3 Depression*

|  | Model 1 |  | Model 2 |  | Model 3a - Men |  | Model 3b - Women |  |
| --- | --- | --- | --- | --- | --- | --- | --- | --- |
|  | OR [%95 CI] |  | OR [%95 CI] |  | OR [%95 CI] |  | OR [%95 CI] | p Gender Difference in Model 3 |
| Gender (Female=1) | 1.35 [0.87, 2.09] |  | 1.33 [0.85, 2.08] |  |  |  |  |  |
| Stressor Exposure |  |  | 1.35* [1.06, 1.73] |  | 1.25 [0.83, 1.87] |  | 1.42* [1.04, 1.94] | 0.62 |
| Negative Affect (NSD) |  |  | 1.12 [0.90, 1.38] |  | 1.09 [0.74, 1.59] |  | 1.14 [0.88, 1.48] | 0.85 |
| Affective Reactivity |  |  | 1.31** [1.08, 1.58] |  | 1.16 [0.84, 1.59] |  | 1.50** [1.15, 1.96] | 0.22 |
| Affective Residue |  |  | 1.04 [0.83, 1.30] |  | 1.05 [0.67, 1.63] |  | 1.06 [0.81, 1.38] | 0.97 |

*Note***.** All models covary for age, education, marital status, employment status, and race. ^†^p<.10, *p<.05, **p<.01
